# *MiR-425-5p* accelerated the proliferation, migration, and invasion of ovarian cancer cells via targeting *AFF4*

**DOI:** 10.1186/s13048-021-00894-x

**Published:** 2021-10-22

**Authors:** Zhihui Wu, Jianlin Guo, Ying Zhang, Jianhua Liu, Hongping Ma, Yurong Tang

**Affiliations:** 1 Department of Clinical Laboratory, Fujian Provincial Maternity and Children Hospital, Affiliated Hospital of Fujian Medical University, Fuzhou, 350001 Fujian Province China; 2 Department of Laboratory, Second People’s Hospital, Kashgar Area, Xinjiang, 844000 China; 3 Department of Clinical Laboratory, Tuoli County People’s Hospital, Tacheng, Xinjiang, 834500 Uygur Autonomous Region China; 4 Department of Clinical Laboratory, Beijing Rehabilitation Hospital, Capital Medical University, Beijing, 100144 China; 5 Department of Clinical Laboratory, Children’s Hospital of Xinjiang Uygur Autonomous Region, Urumqi, Xinjiang, 830054 Uygur Autonomous Region China; 6 Laboratory Department of Shengli Oilfield Central Hospital, Dongying, 257100 China

**Keywords:** Ovarian cancer, *miR-425-5p*, *AFF4*, Proliferation, Invasion, Metastasis

## Abstract

**Background:**

Accumulating data have established that microRNAs (miRNAs) play significant regulatory roles in the carcinogenesis and progression of ovarian cancer (OC). *MiR-425-5p* was reported to function in various tumors. However, the roles and underlying mechanism of *miR-425-5p* involvement in OC development and progression are unclear.

**Methods:**

A comprehensive strategy of data mining, computational biology, and real-time polymerase chain reaction was employed to identify the involvement of *miR-425-5p* in OC progression. The effect of *miR-425-5p* on the proliferation, migration, and invasion of OC cells was determined using Cell Counting Kit-8, wound-healing, and Matrigel invasion assays, respectively. Luciferase assay was performed to evaluate the interactions between *miR-425-5p* and *MAGI2-AS3* or *AFF4*.

**Results:**

*miR-425-5p* was significantly up-regulated in OC tissues and cells. The luciferase reporter assay revealed that *miR-425-5p* was negatively regulated by *MAGI2-AS3*. Silencing *miR-425-5p* inhibited the proliferation, migration, and invasion of OC cells in vitro. Bioinformatics analysis and luciferase reporter assay revealed that *AFF4* was the target gene of *miR-425-5p*. Moreover, *AFF4* expression was significantly decreased in OC and was closely related to the good prognosis of patients with OC. *AFF4* overexpression inhibited the proliferation, migration, and invasion of OC cells in vitro. By contrast, silencing *AFF4* promoted the proliferation, migration, and invasion of OC cells in vitro. Finally, *AFF4* suppression rescued the inhibitory effect of silencing *miR-425-5p* on the proliferation, migration, and invasion of OC cells.

**Conclusion:**

To the best our knowledge, this is the first study to demonstrate that *miR-425-5p* overexpression in OC is negatively regulated by *MAGI2-AS3.* Moreover, miR-425-5p promotes the proliferation, migration, and invasion of OC cells by targeting AFF4, suggesting that *miR-425-5p/AFF4* signaling pathway represented a novel therapeutic target for patients with OC.

**Supplementary Information:**

The online version contains supplementary material available at 10.1186/s13048-021-00894-x.

## Introduction

Ovarian cancer (OC) is one of the major malignancies of the female reproductive system, and it has the highest incidence rate among all gynecological malignancies worldwide. The high incidence is associated with the difficulties of efficient early detection of OC because of its asymptomatic early stage [1]. Despite the availability of surgery, chemotherapy, and radiotherapy, the 5-year overall survival rate of OC was not improved in the past decades [2]. Therefore, it is urgent to examine the mechanism underlying OC development and to find new therapeutic targets to improve the clinical outcomes of patients with OC.

Given the advances in high-resolution microarray and massively parallel sequencing technology, emerging evidence indicates that microRNAs (miRNAs) play complex and extensive roles in the development and progression of various cancers [3]. Studies have reported the involvement of *miR-425-5p* in various cancer processes, and it acts as a tumor suppressor or promoter [4, 5]. However, the exact roles and underlying mechanism of *miR-425-5p* function in OC are still unclear.

In this study, the results demonstrated that *miR-425-5p* was upregulated in OC, and it was negatively regulated by *MAGI2-AS3*. Furthermore, AF4/FMR2 family member 4 (AFF4), as a tumor-suppressor gene in OC, was the new target of *miR-425-5p*. Finally, silencing *miR-425-5p* suppressed the proliferation, migration, and invasion of OC by targeting upregulated *AFF4*.

## Materials and methods

### Data sources

*MAGI2-AS3* expression data in OC were acquired from the online databases of Gene Expression Profiling Interactive Analysis (GEPIA, http://gepia.cancer-pku.cn/) and Gene Expression Omnibus (GEO, https://www.ncbi.nlm.nih.gov/geo/) under accession numbers GSE54388 [6] and GSE74448 [7]. The miRNA expression data in OC were obtained from the GEO under accession numbers GSE83693 [8] and GSE119055 [9]. The expression and prognostic analysis of the mRNAs in OC were obtained from GEPIA and Kaplan–Meier plotter (http://kmplot.com/analysis/index.php). All original data were normalized and log2-transformed. Differentially expressed genes were identified on the basis of | log2 (fold change) | > 1.0 and *p* < 0.05. R software version 3.4.4 (R Foundation for Statistical Computing, Vienna, Austria) was used for all statistical analyses and plotting.

### Identification of *miR-425-5p*

The miRcode is a comprehensive searchable map of putative microRNA target sites in long non-coding RNAs (lncRNAs) [10]. The prediction of target miRNAs of *MAGI2-AS3* was carried out through miRcode (http://www.mircode.org/). Moreover, differentially expressed miRNAs [| log2 (fold change) | > 1 and p < 0.05] between normal and OC tissues were obtained from the GEO under the accession number GSE83693. Subsequently, the intersections of the predicted miRNAs and upregulated miRNAs in OC were obtained as target miRNAs of *MAGI2-AS3*. Finally, the expressions of these miRNAs were further validated using GSE119055.

### Integrated target prediction of *miR-425-5p*

The target prediction of miRNA was performed through TargetScan (http://www.targetscan.org/), Pictar (https://pictar.org/), miRanda (http://www.microrna.org/), microT (http://diana.cslab.ece.ntua.gr/microT/), and miRmap (http://mirnamap.mbc.nctu.edu.tw/). In this study, the intersections of the five databases were recognized as the potential target genes of *miR-425-5p*. These genes were identified for further analysis.

### Correlation analysis

To analyze the correlations of lncRNA–miRNA, lncRNA–mRNA, and miRNA–mRNA pairs in OC, datasets of lncRNA, miRNA, and mRNA expression in OC samples were downloaded from The Cancer Genome Atlas (TCGA, https://cancergenome.nih.gov/) database. The correlation coefficient was calculated using Spearman’s correlation coefficient analysis. The correlation analysis was performed using corrplot R package, and the visualization was made with ggpubr package in R software. The r > 0 and *p* < 0.05 were considered significant positive correlation, whereas r < 0 and *p* < 0.05 were considered significant negative correlation.

### Cell culture

Human OC cell lines (SKOV-3, A2780, and ES-2) and SV-40 transformed human ovarian epithelial cells (IOSE-80) were purchased from the American Type Culture Collection (ATCC, VA, USA). All cells were cultured in Roswell Park Memorial Institute 1640 medium (Gibco; Thermo Fisher Scientific, Inc., Waltham, MA, USA) supplemented with 10% fetal bovine serum (FBS; Gibco, Thermo Fisher Scientific) and 1% penicillin/streptomycin (Euroclone S.p.A, Italy) in an incubator at 37 °C and 5% CO_2_.

### Cell transfection

The pcDNA-MAGI2-AS3 vector (*MAGI2-AS3*), *miR-425-5p* mimics, *miR-425-5p* inhibitor, pcDNA-AFF4 vector (AFF), and *AFF4* shRNAs and their negative control (NC) were obtained from GenePharma, Shanghai, China. pcDNA plasmid without *MAGI2-AS3* or *AFF4* was used as a NC. The sequences of the miRNA mimic, miRNA inhibitor, *AFF4* shRNAs, and their controls are presented in Table S1. Transfection was conducted by using Lipofectamine™ 2000 (Invitrogen; Thermo Fisher Scientific) following the manufacturer’s instructions. After transfection, real-time quantitative polymerase chain reaction (RT-qPCR) analysis was performed to assess the transfection efficiency of these genes.

### RT-qPCR analysis

Total RNA was extracted from cells by using TRIzol reagent (Invitrogen; Thermo Fisher Scientific). cDNA synthesis and RT-qPCR assay for *miR-425-5p* were performed using Hairpin-itTM Real-Time PCR miRNAs kit (GenePharma, Shanghai, China). cDNA syntheses for mRNA and lncRNA were completed using the PrimeScript™ RT reagent Kit with gDNA Eraser (Takara, Dalian, China), and these cDNA samples were amplified by TB Green® Fast qPCR Mix (Takara) in ABI 7500 Fast Real-Time PCR system (Applied Biosystems, Thermo Fisher Scientific). β-actin was used as an internal control for mRNAs and lncRNAs, while U6 snRNA was used as an internal control for miRNAs. RT-qPCR was performed in triplicate, and relative gene expression was analyzed by the 2^-ΔΔCt^ method. The sequences of all primers used in this study are presented in Table S2.

### Cell Counting Kit-8 (CCK-8) assay

The cell viability was assessed by the CCK-8 assay kit (MilliporeSigma, MA, USA) following the manufacturer’s protocol. Briefly, cells were cultured in 96-well plates at a density of 5 × 104 cells per well. Consequently, 10 μL of the fresh CCK-8 solution was added to each well and then co-incubated for 4 h at 37 °C. Finally, the optical density of each well was measured at 450 nm using a microplate reader (Bio-Rad, CA, USA).

### Wound-healing assay

The cell migration capacity was assessed by the wound-healing assay. Briefly, cells were seeded into 6-well plates and incubated for 24 h to allow attachment. The cell layer was scratched with a 20-μL pipette tip, and detached cells were removed carefully by washing with phosphate-buffered saline. The wound area was observed and photographed under optical microscopy (Leica, Germany) after 24 h.

### Transwell invasion assay

The cell invasion capacity was examined using Transwell invasion assay. Briefly, the upper Transwell chambers were pre-coated with Matrigel (BD Biosciences, NJ, USA). The transfected cells were then seeded into the upper chambers that contained a serum-free medium, whereas the lower chambers were filled with medium supplemented with 10% FBS. Furthermore, the Transwell chambers were incubated for 24 h at 37 °C. After the incubation, invasive cells were fixed with 4% paraformaldehyde (Sigma-Aldrich, MO, USA) and then stained with 0.5% crystal violet solution. Finally, migratory cells were counted by optical microscopy (Leica, Germany).

### Luciferase reporter assays

To construct the luciferase reporter vector, *MAGI2-AS3* and *AFF4* 3′-untranslated region (3’UTR) wild-type (WT) fragments containing the binding site of *miR-425-5p* were inserted into the pMir GLO plasmid, while *MAGI2-AS3* and *AFF4* 3’UTR mutant (MUT) fragments were constructed and inserted into the pGL3 pMir GLO plasmid. Briefly, cells were co-transfected with *miR-425-5p* mimics or miR-NC and WT or MUT vectors using Lipofectamine™ 2000 (Invitrogen; Thermo Fisher Scientific). After transfection for 48 h, cells were fully lysed with passive lysis buffer, and luciferase activity was then detected using a dual-luciferase reporter assay system (Promega, Madison, WI, USA) according to the manufacturer’s instructions. The relative luciferase activity was normalized against the Renilla luciferase activity.

### Western blotting analysis

Total protein extracted from cells was placed in radioimmunoprecipitation assay buffer (Vazyme Biotech Co., Ltd., Nanjing, China) supplemented with protease inhibitor cocktail (Roche, Basel, Switzerland), and protein concentrations were measured with the protein BCA assay kit (Invitrogen; Thermo Fisher Scientific). An equal amount of total protein was separated by 10% sodium dodecyl sulfate–polyacrylamide gel electrophoresis (Vazyme Biotech Co., Ltd.) and transferred to polyvinylidene fluoride membrane (Invitrogen; Thermo Fisher Scientific). After blocking with 5% nonfat dried milk, the membrane was incubated with primary antibody against *AFF4* (1:1000, Cell Signaling Technology, MA, USA) and β-action (1:5000, Cell Signaling Technology), followed by horseradish peroxidase-conjugated secondary antibody. Protein detection was visualized with the enhanced chemiluminescent detection reagent (Pierce; Thermo Fisher Scientific) in LI-COR imaging system (LI-COR Biosciences, NE, USA). Protein expression levels were quantified by densitometry using Image J software (National Institutes of Health, Bethesda, MD, USA) and normalized to β-action.

### Statistical analysis

All results were presented as the mean ± standard error of the mean. Statistical analysis was performed by GraphPad Prism 5.0 (GraphPad Software Inc., CA, USA). Student’s t-test was used to compare the difference between the two groups, and one-way analysis of variance test was performed to compare more than two groups; *p* < 0.05 was considered significant.

## Results

### *MiR-425-5p* overexpression in OC was negatively regulated by *MAGI2-AS3*

**Fig. 1:**
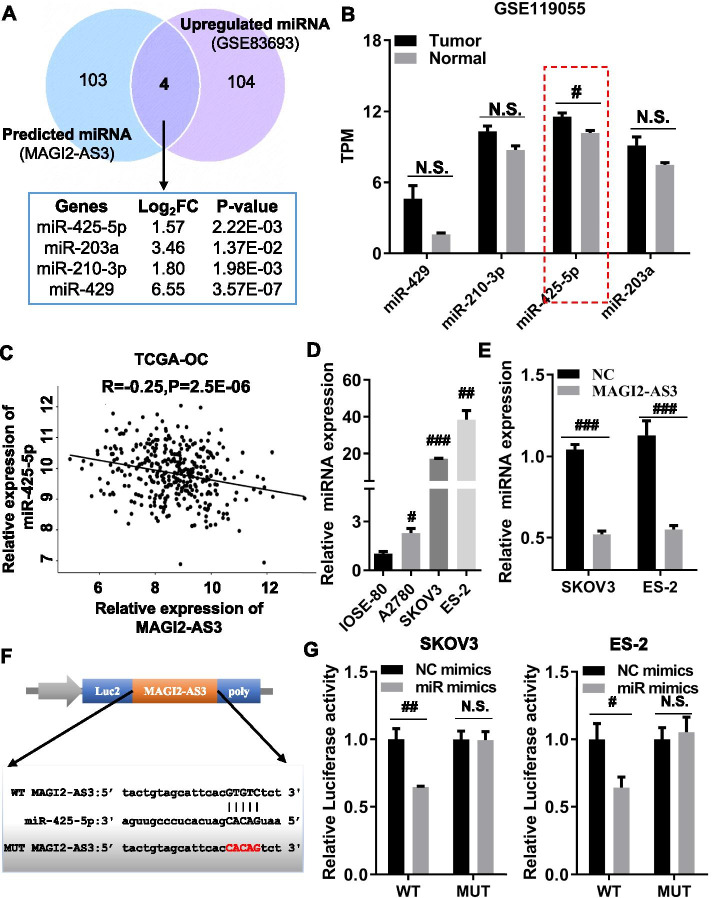
*miR-425-5p* overexpression in ovarian cancer is negatively regulated by *MAGI2-AS3*. **A** Venn diagram showing the overlapping of the predicted target miRNAs and upregulated miRNAs in GSE83693. GSE83693 contained four normal ovarian tissue samples and 16 ovarian cancer samples. **B** GSE119055 datasets were used to verify the expression levels of four miRNAs in ovarian cancer tissues and normal ovarian tissues. GSE119055 contained three normal ovarian tissue samples and six ovarian cancer samples. **C** Correlation of *MAGI2-AS3* and *miR-425-5p* expression. **D***miR-425-5p* expression was detected by real-time quantitative polymerase chain reaction (RT-qPCR) in ovarian cancer cells (A2780, SKOV3, and ES-2) and SV-40 transformed human ovarian epithelial cells (IOSE-80) cells. **E***MiR-425-5p* expression levels were determined by RT-qPCR in SKOV-3 and ES-2 cells, after transient transfection of *MAGI2-AS3* and its negative control (NC). **F** Schematic diagram showing the binding sites of *miR-425-5p* and *MAGI2-AS3* 3’UTR and the mutation site *MAGI2-AS3* 3’UTR. **G** Relative luciferase activities were detected by dual-luciferase reporter assays in SKOV-3 and ES-2 cells after co-transfection of *miR-425-5p* mimics or NC mimics with wild-type-MAGI2-AS3-luc (WT) and mutant-MAGI2-AS3-luc (MUT). ^*N.S*.^*p* > 0.05, ^#^*p* < 0.05, ^##^*p* < 0.01, ^###^*p* < 0.001

We analyzed *MAGI2-AS3* expression using GEPIA and GEO datasets (GSE54388 and GSE74448) in OC and normal tissues. Our results showed that *MAGI2-AS3* expression was significantly downregulated in OC tissues compared with that in normal tissues (Fig. S1A). *MAGI2-AS3* was also detected in three OC cell lines (A2780, SKOV3, and ES-2) and IOSE-80 cells and the results demonstrated that *MAGI2-AS3* expression was significantly lower in A2780, SKOV3, and ES-2 cells than in IOSE-80 cells (Fig. S1B). Then, miRcode was used to predict the potential binding targets of *MAGI2-AS3* and GSE83693 datasets to analyze significantly upregulated miRNAs in OC tissues. Four potential target miRNAs, namely, miR-429, miR-203, miR-210, and miR-425-5p*,* were obtained by integrating miRcode prediction miRNAs and upregulated miRNAs of GSE83693 datasets (Fig. 1A). Further, GSE119055 datasets were selected to validate the expression of the four miRNAs. The results presented that only *miR-425-5p* displayed significant upregulation in OC tissues compared with that in ovarian normal tissues (Fig. 1B). The Spearman correlation coefficient analysis revealed that *miR-425-5p* expression was negatively correlated with *MAGI2-AS3* expression in OC (Fig. 1C). Meanwhile, RT-qPCR results demonstrated that *miR-425-5p* was upregulated in OC cell lines (A2780, SKOV3, and ES-2) compared with that in IOSE-80 cells (Fig. 1D). *MAGI2-AS3* overexpression could reduce *miR-425-5p* expression in SKOV3 and ES-2 cells (Fig. S1C, Fig. 1E). Furthermore, bioinformatics analyses presented the potential binding sites between *miR-425-5p* and *MAGI-AS3* (Fig. 1F). The luciferase reporter assay demonstrated that *miR-425-5p* overexpression significantly suppressed the luciferase activity in WT *MAGI2-AS3* 3’UTR vector but not in *MAGI2-AS3* 3’UTR MUT vector in SKOV3 and ES-2 cells (Fig. 1G), indicating the direct interactions between *miR-425-5p* and *MAGI2-AS3*. Taken together, our data supported the finding that an abnormally high level of *miR-425-5p* expression in OC was directly negatively regulated by *MAGI2-AS3*.

### Silencing *miR-425-5p* suppressed the proliferation, migration, and invasion of OC cells

**Fig. 2:**
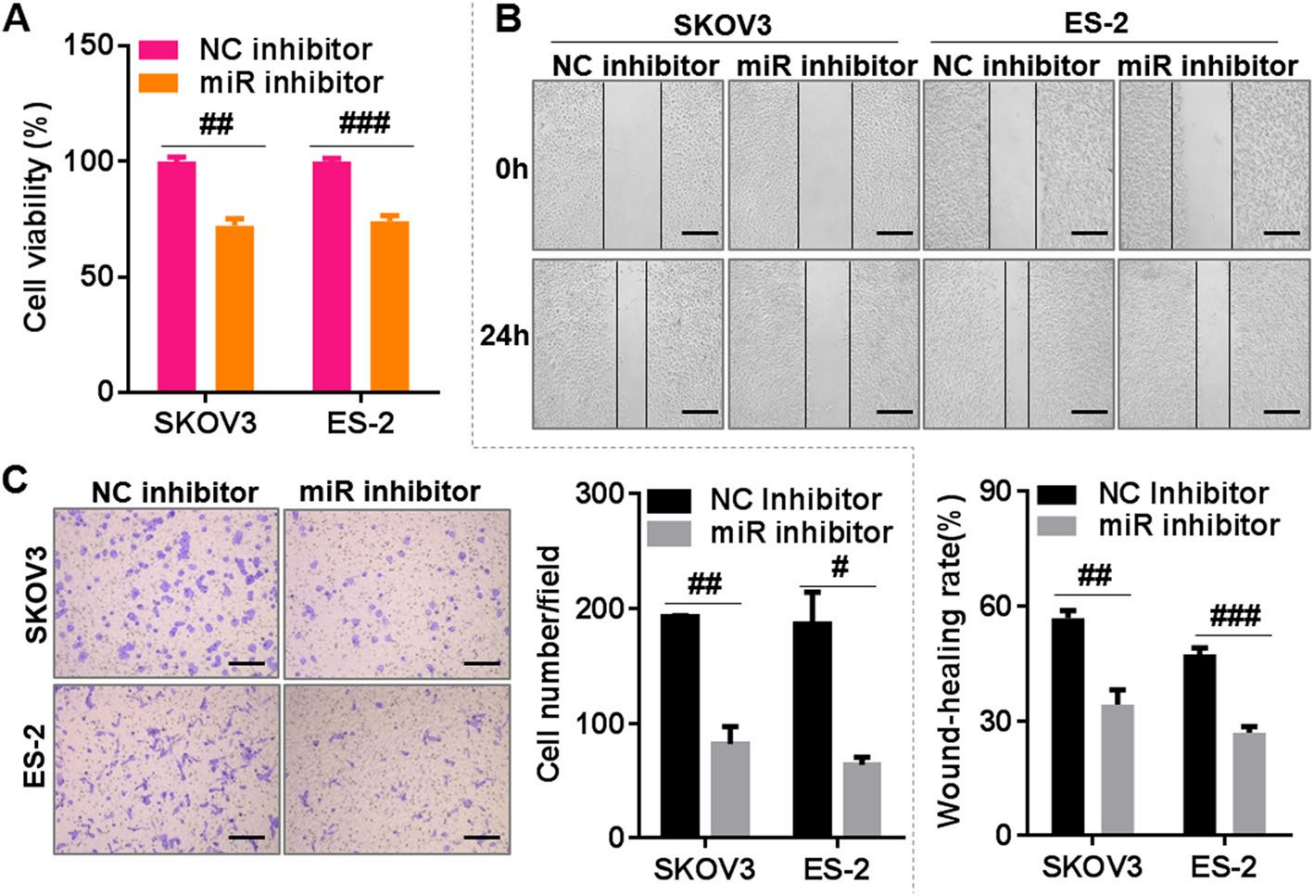
Knockdown of *miR-425-5p* inhibits the proliferation, migration, and invasion of ovarian cancer cells. **A** Cell proliferation capacity was measured using Cell Counting Kit-8 assays in *miR-425-5p* inhibitor (miR inhibitor), or its negative control inhibitor (NC inhibitor), in transfected SKOV-3 and ES-2 cells. **B** Cell migration capacity was measured using wound-healing assays in *miR-425-5p* inhibitor (miR inhibitor), or its NC inhibitor, in transfected SKOV-3 and ES-2 cells. **C** Cell invasion capacity was measured using Transwell assays in *miR-425-5p* inhibitor (miR inhibitor), or its NC inhibitor, in transfected SKOV-3 and ES-2 cells. Bar = 100 μm. ^#^*p* < 0.05, ^##^*p* < 0.01, ^###^*p* < 0.001

To further investigate the role of *miR-425-5p* in OC progression, the *miR-425-5p* inhibitor or NC inhibitor was transfected into OC cells, namely, SKOV3 and ES-2 by using Lipofectamine™ 2000, respectively. The suppression efficiency of the *miR-425-5p* inhibitor after transfection into SKOV3 and ES-2 cells was confirmed by RT-qPCR, and the results indicated that *miR-425-5p* expression was significantly downregulated in OC cells (SKOV3 and ES-2) treated with the *miR-425-5p* inhibitor compared with that in OC cells (SKOV3 and ES-2) treated with the NC inhibitor (Fig. S2). Then, the results of the CCK-8 assays indicated that silencing *miR-425-5p* markedly suppressed the cell proliferation capacity of SKOV3 and ES-2 cells (Fig. 2A). The results of the wound-healing assay suggested that silencing *miR-425-5p* significantly inhibited the migration of SKOV3 and ES-2 cells (Fig. 2B). Meanwhile, the results of the Transwell invasion assay indicated that silencing *miR-425-5p* significantly decreased the invasive abilities of SKOV3 and ES-2 cells (Fig. 2C). These results indicated that silencing *miR-425-5p* could inhibit the malignant phenotype of OC cells, thus confirming the oncogenic role of *miR-425-5p* in OC cells.

### *AFF4*, as a new target of *miR-425-5p*, inhibited the proliferation, migration, and invasion of OC cells

**Fig. 3:**
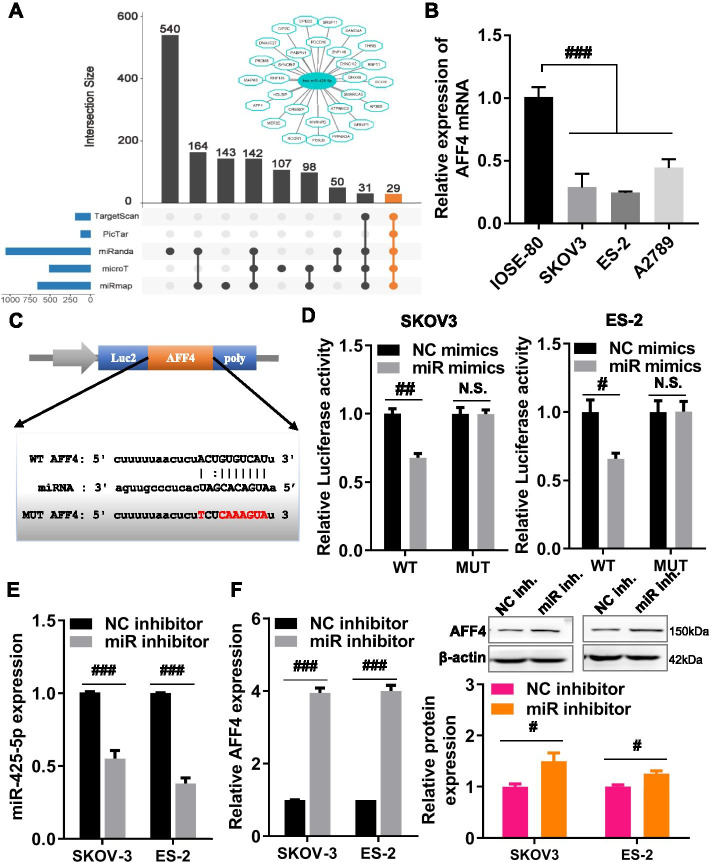
*AFF4* is a direct target of *miR-425-5p* in ovarian cancer cells. **A** To predict potential targets of *miR-425-5p* using multiple miRNA-target prediction tools, the *miR-425-5p*–target interaction network was visualized using Cytoscape 3.7.1. **B***AFF4* expression in ovarian cancer cells (A2780, SKOV3, and ES-2) and IOSE-80 cells was detected by real-time quantitative polymerase chain reaction (RT-qPCR). **C** Schematic diagram showing the binding sites of *miR-425-5p* and *AFF4* 3’UTR and the mutation site of *AFF4* 3’UTR. **D** Relative luciferase activities were detected by dual-luciferase reporter assays in SKOV-3 and ES-2 cells after co-transfection of *miR-425-5p* mimics (miR mimics) or negative control mimics (NC mimics) with wild-type-AFF4-luc (WT) and mutant-AFF4-luc (MUT). **E***MiR-425-5p* expression in SKOV-3 and ES-2 cells was determined by RT-qPCR, after transient transfection of *miR-425-5p* inhibitor (miR inhibitor) and negative control inhibitor (NC inhibitor). **F** The mRNA and protein expressions of *AFF4* in SKOV-3 and ES-2 cells were determined by RT-qPCR and western blot analysis, after transient transfection of *miR-425-5p* inhibitor (miR inhibitor) and NC inhibitor. miR inhibitor, *miR-425-5p* inhibitor; NC inhibitor, corresponding negative control of the *miR-425-5p* inhibitor; miR mimics, *miR-425-5p* mimics; NC mimics, negative control of *miR-425-5p* mimics. ^N.S.^*p* > 0.05, ^#^*p* < 0.05, ^##^*p* < 0.01, ^###^*p* < 0.001

To understand further the molecular mechanisms of *miR-425-5p* in OC, we predicted the potential targets of *miR-425-5p* using five recognizable and promising miRNA target prediction tools, namely, TargetScan, PicTar, miRanda, microT, and miRmap. The results demonstrated that 29 mRNAs are shared in these five tools (Fig. 3A). Further, to understand the roles of these 29 genes in OC, we used GEPIA to analyze the expression patterns of these genes in OC and normal tissues. The results indicated that only 10 of 29 genes were significantly dysregulated in OC samples compared with that in normal ovarian samples (Fig. S3A). Subsequently, the correlations between these 10 dysregulated genes and *miR-425-5p* were analyzed using the TCGA database of OC. The results indicated that *miR-425-5p* was significantly negatively correlated with only 4 of 10 dysregulated genes, including *AFF4*, *MEF2C*, *PRDM8*, and *DIP2C* (Fig. S3B). Based on the miRNA sponge hypothesis, these four genes might be promising targets of *miR-425-5p* (Fig. S3C). Further, the prognostic values of these four genes in OC were determined by Kaplan–Meier plotter, and the results exposed that the expression levels of *MEF2C* and *DIP2C* were not associated with the overall survival of patients with OC, and the high *PRDM8* expression was negatively correlated with the overall survival of patients with OC, while *AFF4* exhibited the opposite correlation: the high *AFF4* expression indicated a better prognosis (Fig. S4). The comprehensive analysis of the above expression analysis, survival analysis, and correlation analysis suggested that *AFF4* played a vital role in the pathogenesis of OC and might be the most promising target gene of *miR-425-5p*.

To determine whether *AFF4* was the target gene of *miR-425-5p* in OC, the mRNA expression of *AFF4* was examined in several OC cell lines (A2780, SKOV3, and ES-2) and IOSE-80 cells by RT-qPCR. The results revealed that the mRNA expression of *AFF4* was significantly downregulated in A2780, SKOV3, and ES-2 cells compared with that in IOSE-80 cells (Fig. 3B). Bioinformatics analysis suggested the potential binding sites of *miR-425-5p* in *AFF4* 3′-UTR (Fig. 3C). To further validate whether *miR-425-5p* directly targets the *AFF4* 3′-UTR by a dual-luciferase reporter gene assay, the WT or MUT *AFF4* 3′-UTR dual-luciferase plasmids and *miR-425-5p* mimics or NC mimics were co-transfected into OC cells. As a result, the *miR-425-5p* overexpression significantly suppressed the luciferase activity of the WT *AFF4* 3’UTR reporter vector but not the MUT *AFF4* 3’UTR reporter vector in SKOV-3 and ES-2 cells (Fig. 3D). In addition, silencing of the *miR-425-5p* expression significantly upregulated the mRNA and protein expression of *AFF4* in SKOV3 and ES-2 cells (Fig. 3E, F), suggesting that *miR-425-5p* negatively regulated *AFF4* expression in SKOV3 and ES-2 cells. Altogether, our results indicated that *miR-425-5p* binds directly to the 3’UTR of *AFF4* to inhibit its expression in OC cells.

**Fig. 4:**
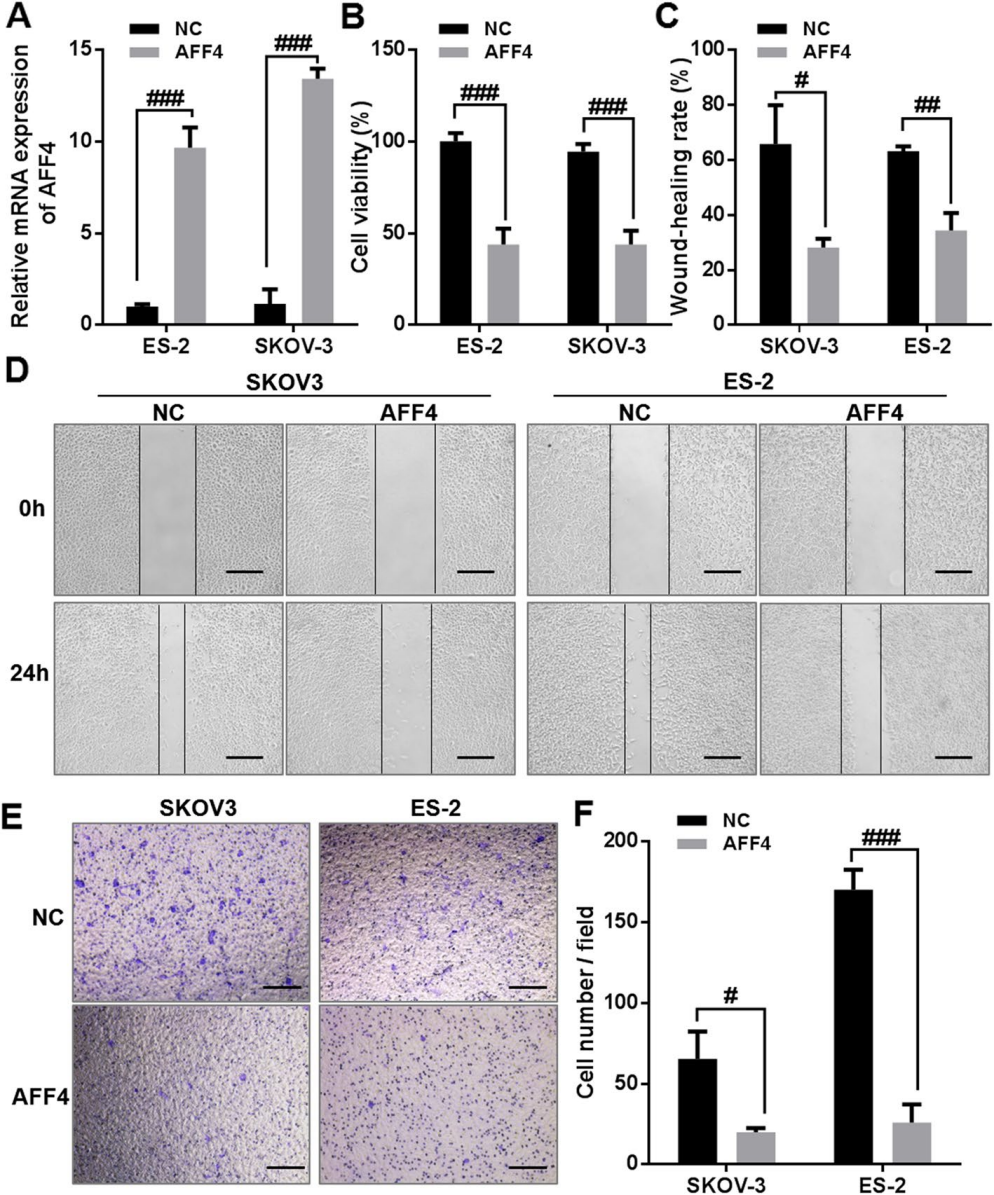
*AFF4* overexpression inhibits the proliferation, migration, and invasion of ovarian cancer cells. **A** AFF4 mRNA expression was determined by a real-time quantitative polymerase chain reaction in SKOV-3 and ES-2 cells, after transient transfection of the overexpression AFF4 vector (AFF4) or its negative control vector (NC). **B** Cell proliferation capacity was measured using Cell Counting Kit-8 assays in the overexpression AFF4 vector (AFF4), or its negative control vector (NC), in transfected SKOV-3 and ES-2 cells. **C**, **D** Cell migration capacity was measured using wound-healing assays in the overexpression AFF4 vector (AFF4), or its negative control (NC) vector, in transfected SKOV-3 and ES-2 cells. **E**, **F** Cell invasion capacity was measured using Transwell assays in the overexpression AFF4 vector (AFF4), or its NC vector, in transfected SKOV-3 and ES-2 cells. Bar = 100 μm. ^#^*p* < 0.05, ^##^*p* < 0.01, ^###^*p* < 0.001

To further investigate the function of *AFF4* in OC, SKOV-3 and ES-2 cells were transfected with overexpression *AFF4* vector (*AFF4*) or its NC vector. The results presented that the mRNA expression of *AFF4* significantly was upregulated in SKOV-3 and ES-2 cells transfected with *AFF4* compared with that in NC-treated SKOV-3 and ES-2 cells (Fig. 4A). The results of the CCK-8 assays indicated that *AFF4* overexpression suppressed the proliferation of SKOV-3 and ES-2 cells (Fig. 4B). The results of the wound-healing assay indicated that *AFF4* overexpression significantly inhibited the migration ability of SKOV-3 and ES-2 cells (Fig. 4C, D). Meanwhile, the results of the Transwell invasion assay indicated that *AFF4* overexpression significantly reduced the invasive ability of SKOV-3 and ES-2 cells (Fig. 4E, F). Altogether, these data indicated that *AFF4* overexpression could inhibit the malignant phenotype of OC cells, suggesting the antioncogenic role of *AFF4* in OC cells.

### Silencing *miR-425-5p* suppressed the malignant phenotype of OC cells by upregulating *AFF4* expression

**Fig. 5:**
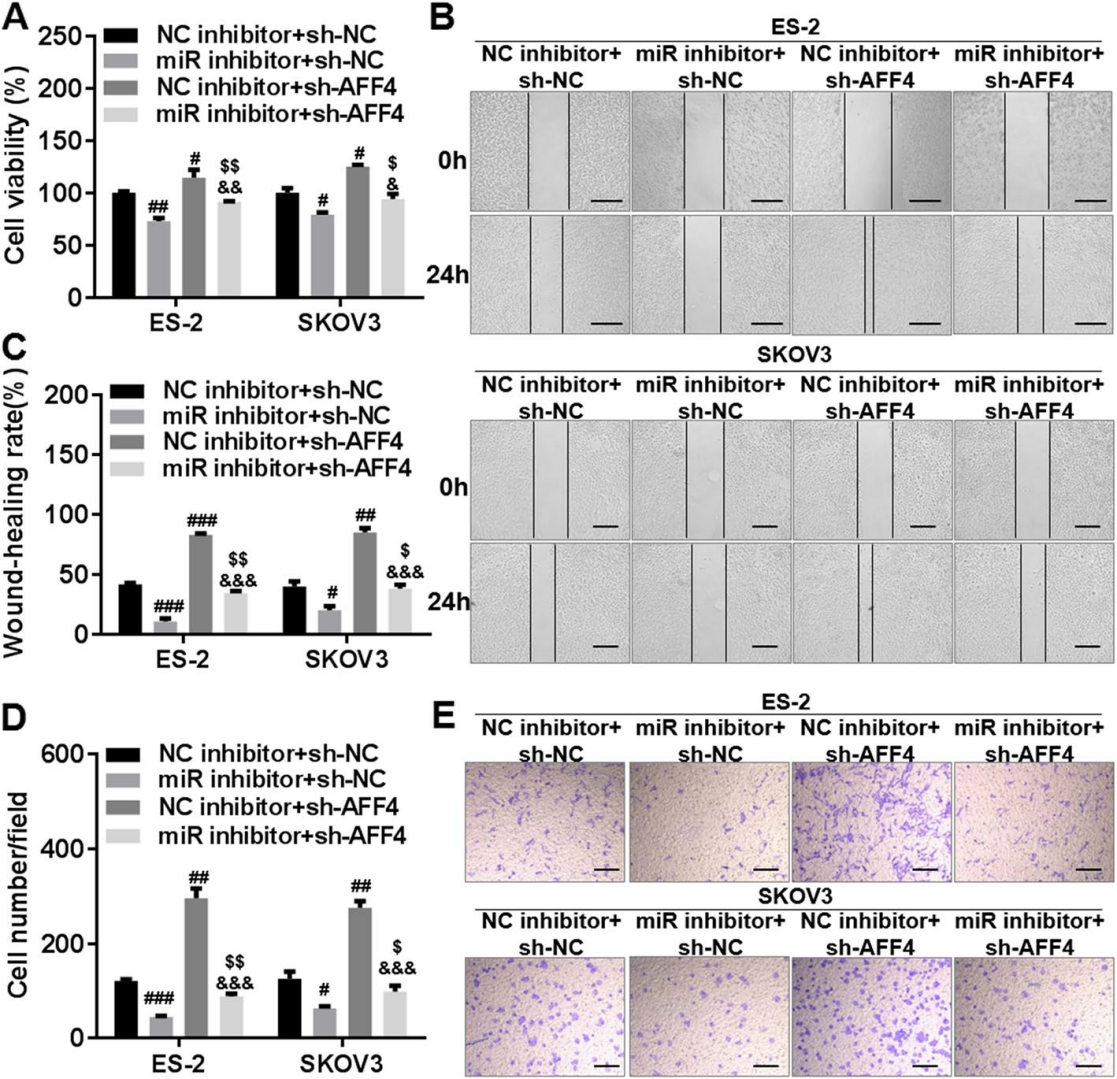
Silencing AAF4 against *miR-425-5p* affects the proliferation, invasion, and migration of ovarian cancer cells. **A** The Cell Counting Kit-8 assay was used to detect cell proliferation in transfected SKOV-3 and ES-2 cells. **B**, **C** The wound-healing assay was used to detect cell migrations in transfected SKOV-3 and ES-2 cells. **D**, **E** The Transwell assay was used to detect cell invasions in transfected SKOV-3 and ES-2 cells. Bar = 100 μm. sh-AFF4, *AFF4* small hairpin RNA; sh-NC, negative control of sh-AFF4; miR inhibitor, *miR-425-5p* inhibitor; NC inhibitor, negative control of *miR-425-5p* inhibitor. ^#^*p* < 0.05, ^###^*p* < 0.001, ^$^*p* < 0.05, and ^$$^*p* < 0.01 comparison with miR inhibitor + sh-NC. ^&^*p* < 0.05, ^&&^*p* < 0.01, and ^&&&^*p* < 0.001 comparison with NC inhibitor + sh-AFF4

To further explore whether *miR-425-5p* inhibited malignant phenotypes in OC cells by regulating *AFF4* expression, we transfected the constructed *AFF4* shRNAs (sh-AFF4#1, sh-AFF4#2, and sh-AFF4#3) and their NC (sh-NC) into OC cells and selected the sh-AFF4 with the best inhibitory effect for the subsequent experiments. The results of the RT-qPCR analysis revealed that all *AFF4* shRNAs (sh-AFF4#1, sh-AFF4#2, and sh-AFF4#3) significantly inhibited *AFF4* expression in SKOV-3 and ES-2 cells, and sh-AFF4#1 (sh-AFF4) was the most effective and could be used for subsequent studies (Fig. S5A). Furthermore, the *miR-425-5p* inhibitor or sh-AFF4 was transfected into OC cells separately or co-transfected with Lipofectamine™ 2000 reagent, and the promotion effect of silencing *miR-425-5p* expression on the mRNA and protein expression of *AFF4* in SKOV-3 and ES-2 cells was significantly inhibited by the transfection of *AFF4* shRNA (Fig. S5B, C). The results of the CCK-8 assay indicated that silencing *AFF4* eradicated the inhibitory effect of silencing *miR-425-5p* on the cell proliferation capacity of SKOV-3 and ES-2 cells (Fig. 5A). The results of the wound-healing assay indicated that silencing *AFF4* dramatically weakened the inhibitory effect of silencing *miR-425-5p* on the cell migration capacity of SKOV-3 and ES-2 cells (Fig. 5B, C). Meanwhile, the results of the Transwell invasion assay indicated that silencing *AFF4* dramatically attenuated the inhibitory effect of silencing *miR-425-5p* on the cell invasion capacity of SKOV-3 and ES-2 cells (Fig. 5D, E). Moreover, silencing *AFF4* could promote the proliferation, migration, and invasion of SKOV-3 and ES-2 cells (Fig. 5A–E), suggesting that silencing *AFF4* promoted the malignant phenotype of OC cells. Taken together, these results suggested that silencing *miR-425-5p* inhibited the malignant phenotype of OC cells by upregulating *AFF4* expression.

## Discussion

OC is one of the most common cancers among women with the third highest incidence rate and the highest mortality rate worldwide [2]. This is due to the insidious onset of OC and the lack of specific early diagnosis and effective long-term therapies. Recent studies are focusing on the development of new diagnostic and therapeutic biomarkers to improve the survival rates [11]. To the best of our knowledge, this was the first study to identify that *miR-425-5p* overexpression in OC was negatively regulated by *MAGI2-AS3* and that silencing *miR-425-5p* inhibited the proliferation, migration, and invasion of OC cells by targeting *AFF4*, suggesting that *miR-425-5p/AFF4* signaling may be a novel therapeutic target for patients with OC.

*MAGI2-AS3* plays a critical role in various cancers [12]. In this study, we demonstrated that *MAGI2-AS3* is significantly downregulated in OC tissues and cell lines, consistent with the results of a previous study [13]. *MAGI2-AS3* is considered an important player in high-grade serous ovarian carcinoma, regulating the downstream mRNA expression by sponging miR-15-5p, miR-374a-5p, and miR-374b-5p in the competing endogenous RNA (ceRNA) regulation network [13]. This study have suggested the suppressor role of *MAGI2-AS3* in OC progression. In addition, lncRNAs may act as competing endogenous RNA (ceRNAs) by repressing the expression of miRNAs and further regulating the associated mRNAs [14]. *LINC00511* mediates the effects of *ESR1* on proliferation and invasion of OC through *miR-424-5p* and *miR-370-5p* [15]. LncRNA PTAR promotes epithelial–mesenchymal transition (EMT) and invasion–metastasis in serous OC by competitively binding *miR-101-3p* to regulate *ZEB1* expression [16]. Based on a comprehensive strategy of data mining, computational biology, and RT-PCR, we identified that *miR-425-5p* was significantly upregulated in OC and may be involved in OC development. Recent studies have reported that *miR-425-5p* may be involved in OC progression, but its specific role and regulatory mechanism are unclear [17]. Our results indicated that silencing *miR-425-5p* inhibited the proliferation, migration, and invasion of OC cells, which was consistent with its role in other cancers. For example, *miR-425-5p* is significantly increased in hepatocellular carcinoma (HCC) tissues and is closely related to the poor prognosis of patients with HCC, which as an oncogene promotes the proliferation and migration of HCC cells via *RNF11* or *FOXD3* [18, 19]. In colorectal cancer, *miR-425-5p* promotes cell proliferation, migration, invasion, and EMT by activating the *CTNND1*-mediated β-catenin pathway [20]. In breast cancer, *miR-425-5p* promotes cell growth by target binding to the PTEN 3‘UTR [21]. In lung cancer, *miR-425-5p* overexpression is positively associated with poor prognosis and enhances the proliferation, migration, and invasion of cells [22, 23]. In renal cell carcinoma, studies have shown that *miR-425-5p* is an oncogene in this cancer [24]. The aforementioned studies have suggested that *miR-425-5p* may act as an oncogene in many cancers, and targeting *miR-425-5p* may be a potential method for the treatment of cancer in the future.

*AFF4* (AF4/FMR2), as a core component of the super elongation complex [25], is involved in the progression of cancer, e.g., leukemia [26], head and neck squamous cell carcinoma [27], melanoma [28], bladder cancer [29, 30], and lung cancer [31]. However, the role of *AFF4* in OC development remains unknown. To the best of our knowledge, we are the first to find that *AFF4* was significantly decreased in OC tissues and cells and was closely related to the good prognosis of patients with OC. Further, our results indicated that *AFF4* overexpression inhibited the proliferation, migration, and invasion of OC cells in vitro. By contrast, silencing *AFF4* promoted the proliferation, migration, and invasion of OC cells in vitro, suggesting that *AFF4* played a tumor-suppressive role in OC. However, *AFF4* played a major oncogenic role in several cancers [26–31], in contrast to its role in OC. A possible reason is that *AFF4*, as a transcription factor, mainly activated tumor-suppressor genes or signaling pathways in OC; thus, the specific regulatory mechanism needs to be further explored. Finally, our data demonstrated that *AFF4* was the new target of *miR-425-5p*, and silencing *AFF4* reversed the malignant phenotype-suppressing effects of *miR-425-5p* knockdown in SKOV-3 and ES-2 cells. Herein, we first revealed that *miR-425-5p* demonstrated oncogenic actions by directly downregulating *AFF4* in OC.

However, this study has two main limitations. First, although silencing *miR-425-5p* can inhibit malignant phenotypes in OC cells, we have not verified whether *miR-425-5p* overexpression promotes malignant phenotypes in OC cells. Second, the role of the *miR-425-5p/AFF4* pathway in OC progression has not been further verified in vivo. Thus, the downstream signaling of *AFF4* in OC remains largely unknown. In the further study, these parts in our experiment will be designed.

## Conclusions

The results of this study showed, for the first time, that *miR-425-5p* was significantly upregulated in OC tissues and cells, and it was negatively regulated by *MAGI2-AS3*. Further, *AFF4*, which acts as a tumor-suppressor gene in OC, was the new target for *miR-425-5p*. Finally, silencing *miR-425-5p* suppressed the proliferation, migration, and invasion of OC cells by targeting upregulated *AFF4*. Our findings suggested that *miR-425-5p/AFF4* signaling may be a potential therapeutic target for patients with OC in the future.

## Supplementary Information

**Additional file 1: Figure S1.** *MAGI2-AS3* expression in ovarian cancer tissues and cells. (A) *MAGI2-AS3* was downregulated in ovarian cancer tissues based on Gene Expression Profiling Interactive Analysis and Gene Expression Omnibus. GSE54388 contained six normal ovarian tissue samples and 12 ovarian cancer samples. GSE74448 contained 11 normal ovarian tissue samples and 29 ovarian cancer samples. (B) The level of *MAGI2-AS3* expression in IOSE-80, A2780, SKOV3, and ES-2 cells was detected by real-time quantitative polymerase chain reaction (RT-qPCR). (C) The levels of *MAGI2-AS3* expression in SKOV-3 and ES-2 cells were determined by RT-qPCR, after transient transfection of *MAGI2-AS3* and its negative control. ^#^*p* < 0.05, ^##^*p* < 0.01, ^###^*p* < 0.001.

**Additional file 2: Figure S2.** *MiR-425-5p* expression levels were determined by real-time quantitative polymerase chain reaction in SKOV-3 and ES-2 cells, after transient transfection of *miR-425-5p* mimics and negative control mimics. ^#^*p* < 0.05, ^###^*p* < 0.001.

**Additional file 3: Figure S3.** Identification of potential target genes of *miR-425-5p*. (A) The expressions of 29 target genes in ovarian cancer tissues were analyzed by GEPIA data of ovarian cancer (only showed different target genes). (B) Correlations between *miR-425-5p* and different target genes in ovarian cancer (only showed significant correlation). (C) Overview of genes negatively associated with *miR-425-5p*.

**Additional file 4: Figure S4.** Overall survival rate of four hub targets by Kaplan–Meier survival analysis in ovarian cancer.

**Additional file 5: Figure S5.** Effects of *AFF4* knockdown on *miR-425-5p* regulation. (A) The levels of *AFF4* expression were determined by real-time quantitative polymerase chain reaction (RT-qPCR) in SKOV-3 and ES-2 cells, after transient transfection of *AFF4* shRNA#1 (sh-AFF-1), *AFF4* shRNA#2 (sh-AFF-2), *AFF4* shRNA#3 (sh-AFF-3), and its negative control (sh-NC). (B) The mRNA expression of *AFF4* was determined by RT-qPCR in SKOV-3 and ES-2 cells with different treatments. (C) The protein expression of *AFF4* was determined by western blot analyses in SKOV-3 and ES-2 cells with different treatments. sh-AFF4, *AFF4* small hairpin RNA; sh-NC, negative control of sh-AFF4; miR inhibitor, *miR-425-5p* inhibitor; NC inhibitor, negative control of *miR-425-5p* inhibitor. ^#^*p* < 0.05, ^###^*p* < 0.001. ^$^*p* < 0.05, ^$$^*p* < 0.01 comparison with miR inhibitor + sh-NC. ^&^*p* < 0.05 comparison with NC inhibitor + sh-AFF4.

**Additional file 6: Table S1.** Sequences of *miR-425-5p* mimic, *miR-425-5p* inhibitor, and *sh-AFF4* that were used in the study.

**Additional file 7: Table S2.** Primer sequences of *MAGI2-AS3*, *miR-425-5p*, *AFF4*, *U6* and *GAPDH*.

## Data Availability

The datasets used and/or analysed during the current study are available from the corresponding author on reasonable request.

## Abbreviations

OC: Ovarian cancer
miRNAs: MicroRNAs
AFF4: AF4/FMR2 family member 4
GEPIA: Gene Expression Profiling Interactive Analysis
GEO: Gene Expression Omnibus
LncRNAs: Long non-coding RNAs
TCGA: The Cancer Genome Atlas
RT-qPCR: Real-time quantitative polymerase chain reaction
CCK-8: Cell Counting Kit-8
3’UTR: 3′-untranslated region
ceRNA: Competing endogenous RNA
HCC: Hepatocellular carcinoma
