# Supplementary figures and images for: MiR-425-5p accelerated the proliferation, migration, and invasion of ovarian cancer cells via targeting AFF4
Source: J Ovarian Res. 2021 Oct 22;14:138. doi: 10.1186/s13048-021-00894-x (PMC8539801; doi:10.1186/s13048-021-00894-x)

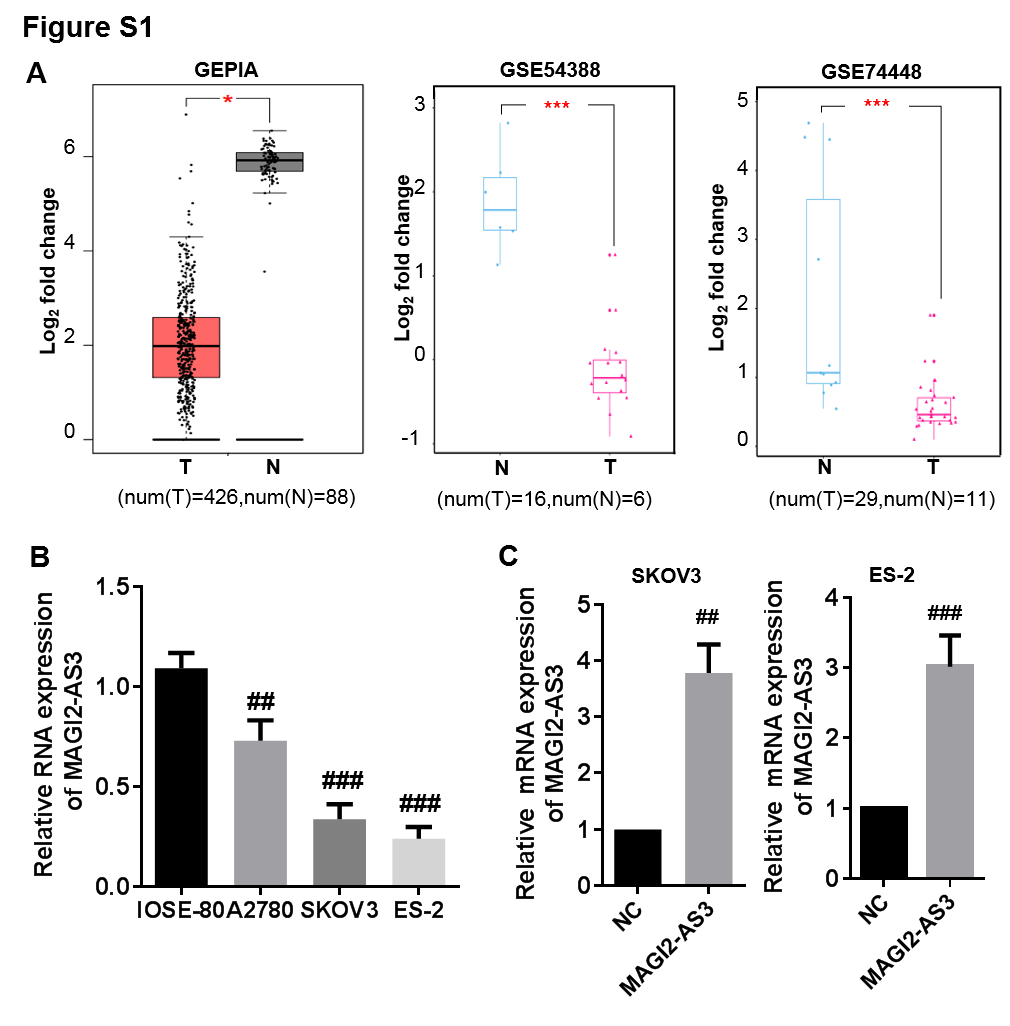

Supplement: Supplementary file 1 — Additional file 1: Figure S1. MAGI2-AS3 expression in ovarian cancer tissues and cells. (A) MAGI2-AS3 was downregulated in ovarian cancer tissues based on Gene Expression Profiling Interactive Analysis and Gene Expression Omnibus. GSE54388 contained six normal ovarian tissue samples and 12 ovarian cancer samples. GSE74448 contained 11 normal ovarian tissue samples and 29 ovarian cancer samples. (B) The level of MAGI2-AS3 expression in IOSE-80, A2780, SKOV3, and ES-2 cells was detected by real-time quantitative polymerase chain reaction (RT-qPCR). (C) The levels of MAGI2-AS3 expression in SKOV-3 and ES-2 cells were determined by RT-qPCR, after transient transfection of MAGI2-AS3 and its negative control. #p < 0.05, ##p < 0.01, ###p < 0.001. [file 13048_2021_894_MOESM1_ESM.tif]

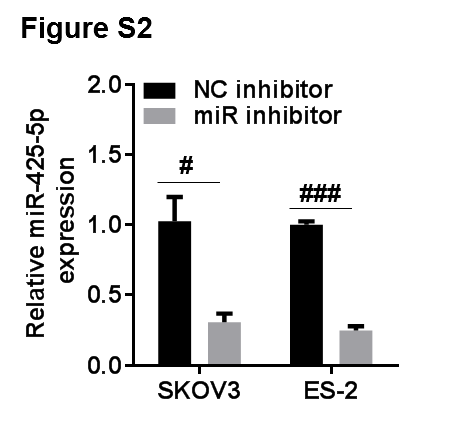

Supplement: Supplementary file 2 — Additional file 2: Figure S2. MiR-425-5p expression levels were determined by real-time quantitative polymerase chain reaction in SKOV-3 and ES-2 cells, after transient transfection of miR-425-5p mimics and negative control mimics. #p < 0.05, ###p < 0.001. [file 13048_2021_894_MOESM2_ESM.tif]

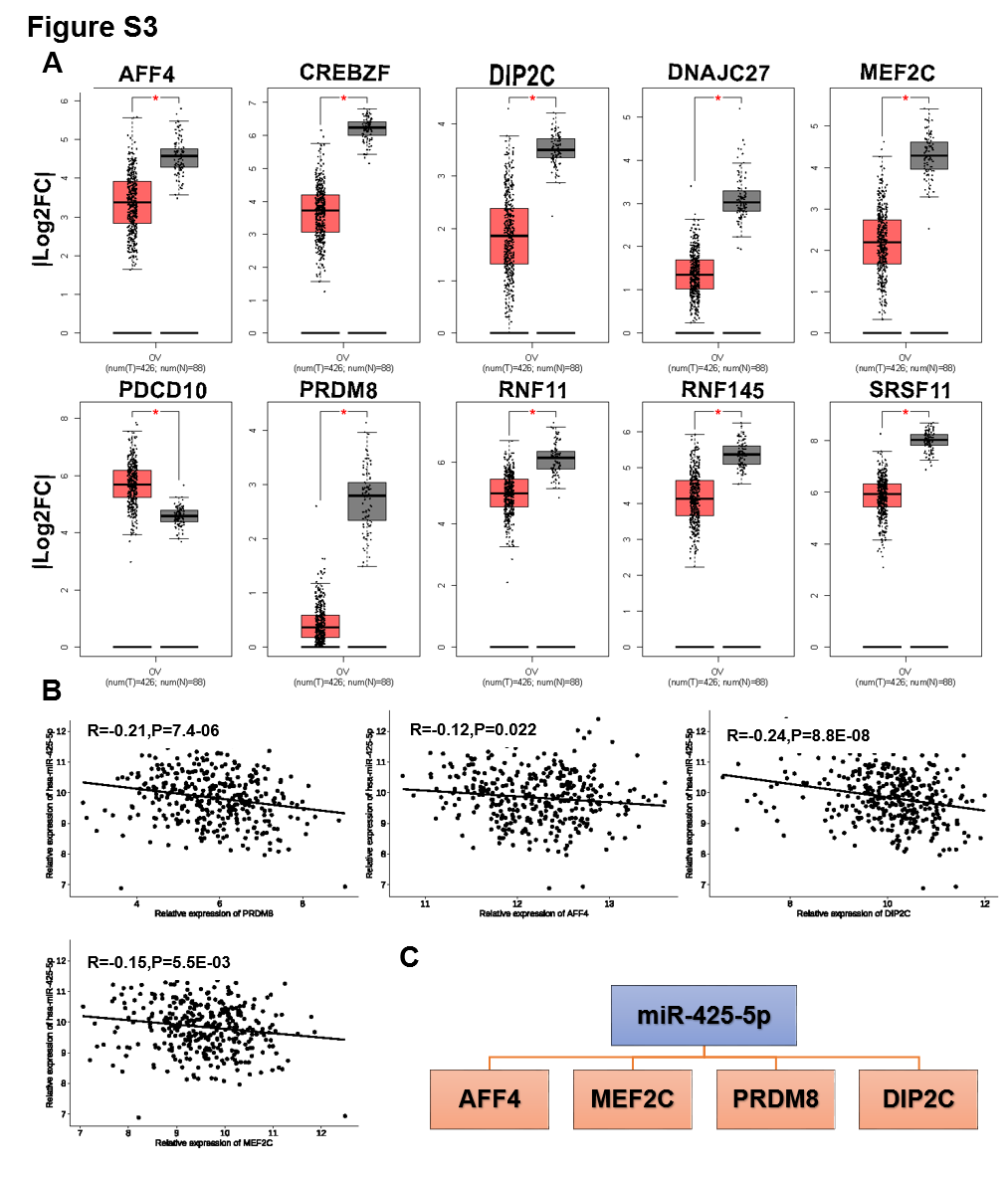

Supplement: Supplementary file 3 — Additional file 3: Figure S3. Identification of potential target genes of miR-425-5p. (A) The expressions of 29 target genes in ovarian cancer tissues were analyzed by GEPIA data of ovarian cancer (only showed different target genes). (B) Correlations between miR-425-5p and different target genes in ovarian cancer (only showed significant correlation). (C) Overview of genes negatively associated with miR-425-5p. [file 13048_2021_894_MOESM3_ESM.tif]

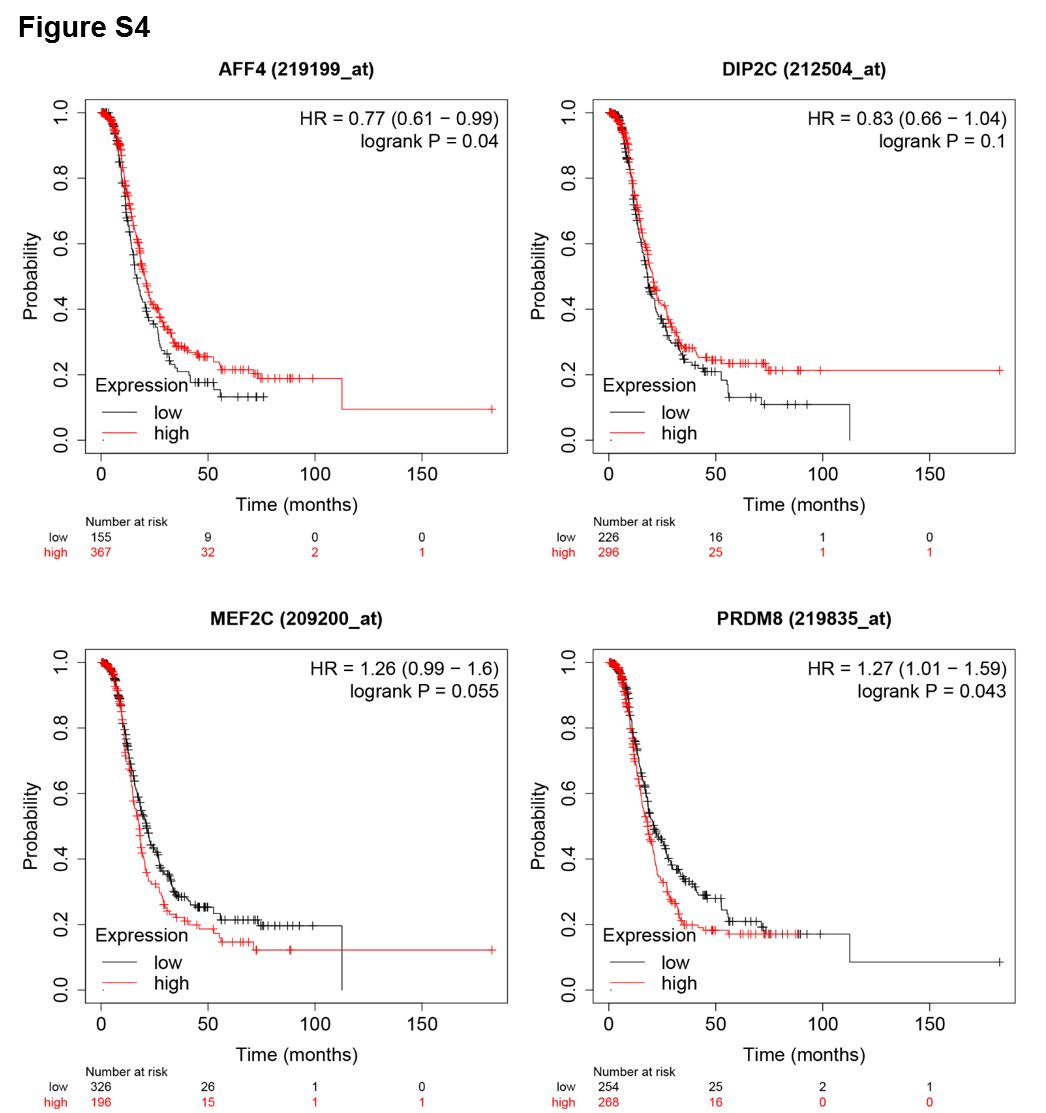

Supplement: Supplementary file 4 — Additional file 4: Figure S4. Overall survival rate of four hub targets by Kaplan–Meier survival analysis in ovarian cancer. [file 13048_2021_894_MOESM4_ESM.tif]

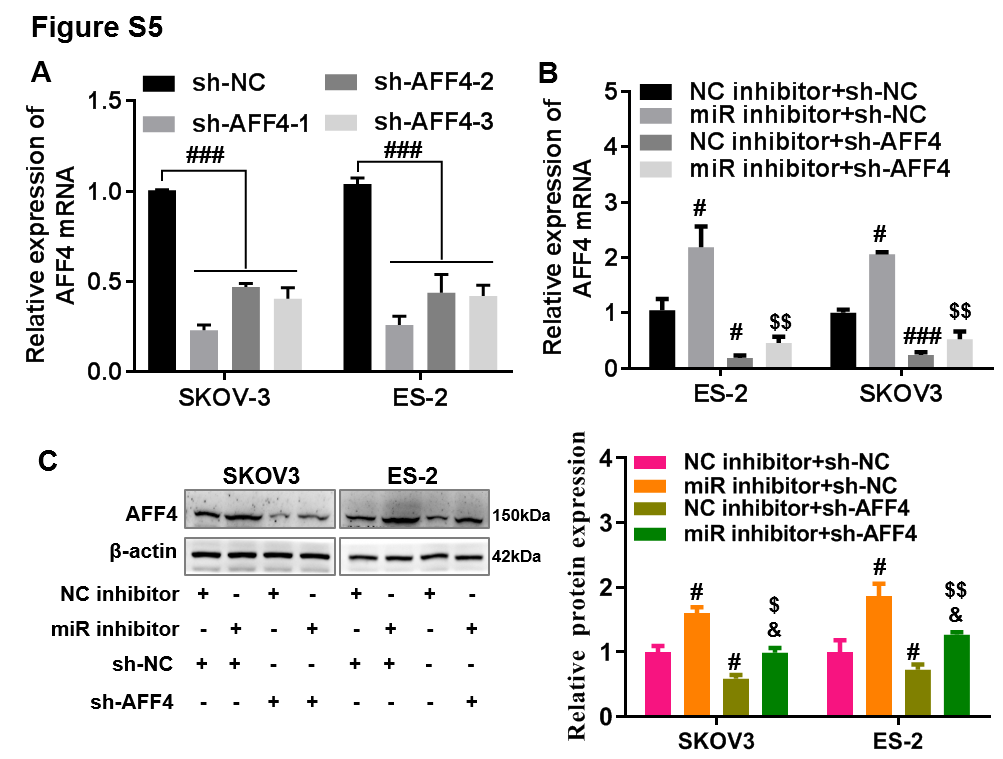

Supplement: Supplementary file 5 — Additional file 5: Figure S5. Effects of AFF4 knockdown on miR-425-5p regulation. (A) The levels of AFF4 expression were determined by real-time quantitative polymerase chain reaction (RT-qPCR) in SKOV-3 and ES-2 cells, after transient transfection of AFF4 shRNA#1 (sh-AFF-1), AFF4 shRNA#2 (sh-AFF-2), AFF4 shRNA#3 (sh-AFF-3), and its negative control (sh-NC). (B) The mRNA expression of AFF4 was determined by RT-qPCR in SKOV-3 and ES-2 cells with different treatments. (C) The protein expression of AFF4 was determined by western blot analyses in SKOV-3 and ES-2 cells with different treatments. sh-AFF4, AFF4 small hairpin RNA; sh-NC, negative control of sh-AFF4; miR inhibitor, miR-425-5p inhibitor; NC inhibitor, negative control of miR-425-5p inhibitor. #p < 0.05, ###p < 0.001. $p < 0.05, $$p < 0.01 comparison with miR inhibitor + sh-NC. &p < 0.05 comparison with NC inhibitor + sh-AFF4. [file 13048_2021_894_MOESM5_ESM.tif]
