# Supplementary material for: MiR-425-5p accelerated the proliferation, migration, and invasion of ovarian cancer cells via targeting AFF4
Source: J Ovarian Res. 2021 Oct 22;14:138. doi: 10.1186/s13048-021-00894-x (PMC8539801; doi:10.1186/s13048-021-00894-x)
Supplement: Supplementary file 6 — Additional file 6: Table S1. Sequences of miR-425-5p mimic, miR-425-5p inhibitor, and sh-AFF4 that were used in the study. [file 13048_2021_894_MOESM6_ESM.docx]

**Supplement tables**

**Table S1.** Sequences of miR-425-5p mimic, miR-425-5p inhibitor and sh-AFF4 were used in the study.

| **genes** | **Sequence (5’-3’)** |
| --- | --- |
| miR-425-5p mimics | AAUGACACGAUCACUCCCGUUGA |
| NC mimics | AUCUGCGAAGGUGUCUCGUTT |
| miR-425-5p inhibitor | UUACUGUGCUAGUGAGGGCAACU |
| NC inhibitor | CAGUACUUUUGUGUAGUACAA |
| AFF4 shRNA#1 | CCGGCAGTTGCAATTCATAACTAATCTCGAGATTAGTTAT  GAATTGCAACTGTTTTTTG |
| AFF4 shRNA#2 | CCGGACTGATACAAGTGGACCTAAACTCGAGTTTAGGT  CCACTTGTATCAGTTTTTTTG |
| AFF4 shRNA#3 | CCGGGTAAGAATTGGTTCGTCTAAACTCGAGTTTAGA  CGAACCAATTCTTACTTTTTTG |
