# Supplementary material for: MiR-425-5p accelerated the proliferation, migration, and invasion of ovarian cancer cells via targeting AFF4
Source: J Ovarian Res. 2021 Oct 22;14:138. doi: 10.1186/s13048-021-00894-x (PMC8539801; doi:10.1186/s13048-021-00894-x)
Supplement: Supplementary file 7 — Additional file 7: Table S2. Primer sequences of MAGI2-AS3, miR-425-5p, AFF4, U6 and GAPDH. [file 13048_2021_894_MOESM7_ESM.docx]

**Supplement tables**

**Table S 2.** Primer sequences of MAGI2-AS3, miR‑425‑5p, AFF4, U6 and GAPDH

| **Genes** | **Sequence (5’-3’)** |
| --- | --- |
| miR-425-5p | RT primer, GTTGGCTCTGGTGCAGGGTCCGAGGTATTCGCACCAGAGCCAACTCAACG  Forward primer, ATGACACGATCACTCCCGTTG  Reverse primer, GTGCAGGGTCCGAGGTATTC |
| MAGI2-AS3 | Forward primer, TGGGTCTGTGCAGAGTTGAG  Reverse primer, GGGAGTCTAGGCCCCTTCTA |
| AFF4 | Forward primer, AAGACTTGGCCTAAGACGTAAA  Reverse primer, TTCCACATTTCGTATCTCTCCC |
| U6 | RT primer, GTTGGCTCTGGTGCAGGGTCCGAGGTATTCGCACCAGAGCCAACAAAATATGG  Forward primer, 5'‑CTCGCTTCGGCAGCACA‑3'  Reverse primer,5'‑AACGCTTCACGAATTTGCGT‑3' |
| GAPDH | Forward primer, 5'‑GCACCGTCAAGGCTGAGAAC‑3'  Reverse primer, 5'‑TGGTGAAGACGCCAGTGGA‑3 |
